# Establishing a generalized polyepigenetic biomarker for tobacco smoking

**DOI:** 10.1038/s41398-019-0430-9

**Published:** 2019-02-15

**Authors:** Karen Sugden, Eilis J. Hannon, Louise Arseneault, Daniel W. Belsky, Jonathan M. Broadbent, David L. Corcoran, Robert J. Hancox, Renate M. Houts, Terrie E. Moffitt, Richie Poulton, Joseph A. Prinz, W. Murray Thomson, Benjamin S. Williams, Chloe C. Y. Wong, Jonathan Mill, Avshalom Caspi

**Affiliations:** 1 Department of Psychology and Neuroscience, Duke University, Durham, NC USA; 2 Center for Genomic and Computational Biology, Duke University, Durham, NC USA; 3 University of Exeter Medical School, University of Exeter, Exeter, UK; 4 Social, Genetic, and Developmental Psychiatry Research Centre, Institute of Psychiatry, Psychology, and Neuroscience, King’s College London, London, UK; 5 Department of Epidemiology & Butler Aging Center, Columbia University Mailman School of Public Health, New York, NY USA; 6 Department of Oral Sciences, University of Otago, Dunedin, New Zealand; 7 Department of Preventive and Social Medicine, Dunedin School of Medicine, University of Otago, Dunedin, New Zealand; 8 Department of Psychiatry and Behavioral Sciences, Duke University School of Medicine, Durham, NC USA; 9 Dunedin Multidisciplinary Health and Development Research Unit, University of Otago, Dunedin, New Zealand

## Abstract

Large-scale epigenome-wide association meta-analyses have identified multiple ‘signatures’’ of smoking. Drawing on these findings, we describe the construction of a polyepigenetic DNA methylation score that indexes smoking behavior and that can be utilized for multiple purposes in population health research. To validate the score, we use data from two birth cohort studies: The Dunedin Longitudinal Study, followed to age-38 years, and the Environmental Risk Study, followed to age-18 years. Longitudinal data show that changes in DNA methylation accumulate with increased exposure to tobacco smoking and attenuate with quitting. Data from twins discordant for smoking behavior show that smoking influences DNA methylation independently of genetic and environmental risk factors. Physiological data show that changes in DNA methylation track smoking-related changes in lung function and gum health over time. Moreover, DNA methylation changes predict corresponding changes in gene expression in pathways related to inflammation, immune response, and cellular trafficking. Finally, we present prospective data about the link between adverse childhood experiences (ACEs) and epigenetic modifications; these findings document the importance of controlling for smoking-related DNA methylation changes when studying biological embedding of stress in life-course research. We introduce the polyepigenetic DNA methylation score as a tool both for discovery and theory-guided research in epigenetic epidemiology.

## Introduction

Tobacco smoking is the greatest health hazard in the modern world. Smoking is associated with practically every risk factor known to impact health and with numerous clinical endpoints^1^. As such, uncovering methods to identify and quantify the biological mechanisms through which tobacco smoking exerts its effects is of critical public health importance.

One such mechanism is DNA methylation. Smoking is known to exact pervasive alterations on the blood epigenome^2–5^. Epigenome-wide association studies (EWAS) of DNA methylation have identified thousands of CpG sites differentially methylated in smokers, though the significance of these discoveries for population health science remains uncertain. If DNA methylation changes accumulate with increased exposure and attenuate with sustained abstinence, DNA methylation analysis could provide clinicians and researchers with a biomarker of smoking-associated health risk.

Some attempt has been made to capture these manifold DNA methylation differences into single, tangible biomarkers describing various aspects of smoking-related behavior and outcomes. At the simplest level, DNA methylation level at a single CpG site within *AHRR* (described by Illumina array probe ID cg05575921) has been shown to discriminate between adult non-smokers and current-smokers^6,7^, and former- from never-smokers^8^, while other multi-probe scores have also been developed to assess various phenotypes, ranging from exposure to maternal smoking^9–11^ to smoking-related mortality^12^. However, the suitability of these scores to index generalized smoking phenotypes is unknown, since these metrics are usually developed and tested in one specific population at one point in time with one specific outcome. Additionally, temporal dynamics of composite DNA methylation metrics cannot be assessed using cross-sectional designs.

Longitudinal repeated-measures analysis of blood DNA methylation in smokers and non-smokers followed over time is needed to test (a) if further accumulation of exposure in smokers is reflected in accumulating changes to DNA methylation, and (b) if cessation and abstinence are associated with reversal of these changes. Finally, data are needed that observe organ system damage caused by smoking in order to test if a DNA methylation biomarker measures not just smoking behavior, but substantive health consequences of tobacco exposure.

Here, we address four major themes relevant to the relationship between tobacco smoking and DNA methylation. First, draw on published EWAS findings of smoking to construct a DNA methylation-based algorithm (Smoking methylation PolyEpigenetic Score, SmPEGS) and test its association with cross-sectional smoking phenotypes. Second, we examine the hypothesis that tobacco smoking has causal effects on DNA methylation in blood via investigation of (a) SmPEGS among twins discordant for smoking, and (b) the pattern of change over time in SmPEGS for those who either increase or cease tobacco consumption, or become nicotine dependent. In addition, we examine the relationship between SmPEGS and gene expression profiles in those who do, and do not, smoke in an attempt to uncover potential mechanistic influences of SmPEGS. Third, we analyze longitudinal repeated-measures data on lung function and periodontal disease to test if smoking-associated DNA methylation could provide a biomarker of biological damage arising from tobacco exposure. Fourth, to demonstrate a potential application of polyepigenetic scores, we conduct EWAS of adverse childhood experiences, an exposure thought to have long-term impacts on health via biological embedding. We examine the confounding effects of tobacco smoking in DNA methylation research and demonstrate the potential of utilizing the SmPEGS in lieu of observational smoking data as a covariate to reduce spurious associations in studies of the effects of early adversity on health.

We address these themes using data from two cohorts. The first is the Dunedin Longitudinal Study, a longitudinal birth cohort of 1037 individuals born in 1972–73 and followed repeatedly until the latest assessment at age-38 years. Study members gave blood for DNA analysis first at age-26, and 12 years later when they were aged 38. The second cohort is the E-Risk longitudinal twin study, a cohort of 2232 twins (56% MZ) born in 1994. E-Risk Study members gave blood for DNA analysis at the most recent assessment, aged 18 years.

## Materials and methods

Detailed information about sampling, measurement, and statistical analysis is available in the Supplementary materials.

### Sample

#### Dunedin Study

Participants were members of the Dunedin Longitudinal Study. Participants (*N* = 1037; 91% of eligible births; 52% male) were all individuals born between April 1972 and March 1973 in Dunedin, New Zealand, who were eligible based on residence in the province and who participated in the first assessment at age 3^13^. The cohort represented the full range of socioeconomic status (SES) in the general population of New Zealand’s South Island. Assessments were carried out at birth and ages 3, 5, 7, 9, 11, 13, 15, 18, 21, 26, 32, and, most recently, 38 years, (95% retention rate).

#### E-Risk Study

The Environmental Risk (E-Risk) Longitudinal Twin Study, tracks the development of a 1994–1995 birth cohort of 2232 British children^14^. Briefly, the E-Risk sample was constructed in 1999 and 2000, when 1116 families (93% of those eligible) with same-sex 5-year-old twins participated in home-visit assessments. This sample comprised 56% monozygotic and 44% dizygotic twin pairs, and sex was evenly distributed within zygosity (49% male). The study sample represents the full distribution of socioeconomic conditions in Great Britain. Assessments were carried out at ages 5, 7, 10, 12, and, most recently, 18 years (93% retention rate).

#### Smoking history

Smoking behavior in Dunedin has been assessed repeatedly between ages 15–38 years, including information about quantity smoked, cessation, and second-hand smoke exposure. In E-Risk, participants were interviewed about their smoking history at age-18 years.

#### DNA methylation

In Dunedin, DNA was derived from peripheral blood drawn at ages 26 and 38 years. In E-Risk, peripheral whole blood was drawn at age-18 years. In both cohorts, DNA methylation was measured using the Illumina Infinium HumanMethylation450 BeadChip (“Illumina 450K array”; Illumina, CA, USA).

#### Gum health

Periodontal attachment loss was assessed at ages 26 and 38 in the Dunedin Study by calibrated dental examiners.

#### Lung function

Single-breath diffusing capacity for carbon monoxide (DLco/VA) was assessed at ages 26 and 38 in the Dunedin Study according to European Respiratory Society/American Thoracic Society standards using a body plethysmograph (CareFusion, Yorba Linda, CA). Measures were corrected for hemoglobin, height, smoking within an hour of assessment and sex^15^.

#### Gene expression

RNA was derived from peripheral blood drawn into PAXGene RNA tubes at age-38 in Dunedin. Expression data were generated from whole-blood RNA using the Affymetrix PrimeView Human Gene Chip (Affymetrix, CA, USA).

#### Adverse childhood experiences (ACEs)

The measured ACEs correspond to the ten subcategories of childhood adversity introduced by the U.S. Centers for Disease Control & Prevention (CDC) Adverse Childhood Experiences Study;^16^ i.e., three types of abuse (emotional, physical, sexual), five types of household challenges (household partner violence, household substance abuse, mental illness in household, loss of a parent due to parental death, separation, or divorce, incarceration of a family member), and two types of neglect (emotional, physical).

### Statistical analysis

All analyses were performed in the R statistical programming environment (version 3.4.2). In brief, linear regression was used to test association between dependent and independent variables, while in E-Risk, Generalized Estimating Equations (GEE) were used to account for the clustering within families.

### Code availability

Code available on request from corresponding author.

## Results

### A. Methylation polygenic scores were differentially distributed in never-, former-, and current-smokers

**Fig. 1:**
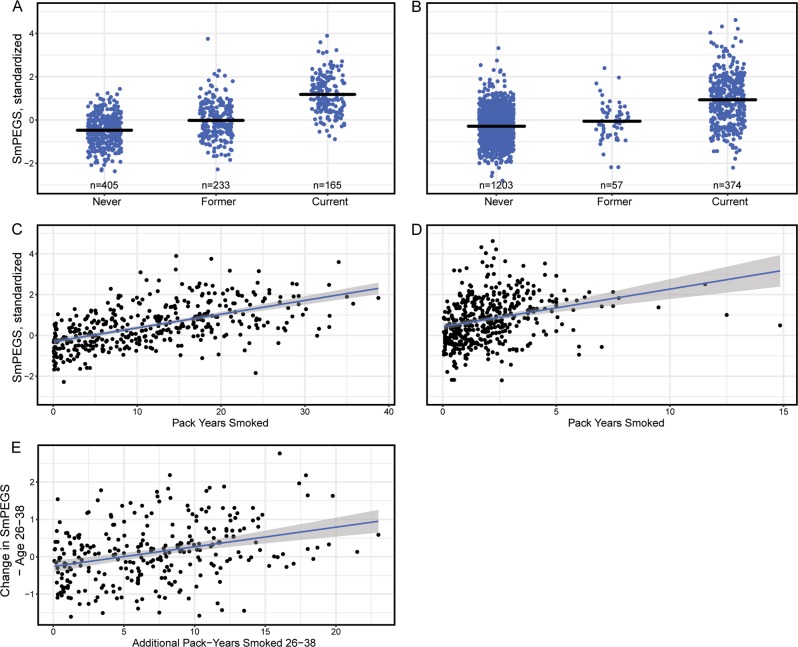
Distribution of SmPEGS across different smoking phenotypes in the Dunedin Study and the E-Risk Study. **a** (Dunedin Study) and **b** (E-Risk Study) show the distribution in never-, former-, and current-smokers. Black bars represent mean values per group. **c** and **d** plot the association of SmPEGS and smoking pack-years in study members who report any smoking in the Dunedin Study (**c**) and the E-Risk Study (**d**). The Dunedin Study members were assessed at age-38, whereas the E-Risk Study members were assessed to age-18; hence their truncated years of potential smoking compared to the Dunedin Study (*x*-axis). **e** plots the association of the change in pack-years smoked and change in SmPEGS in Study members who report any smoking between ages 26 and 38 in the Dunedin Study. SmPEGS = Smoking Methylation Polygenic Score. SmPEGS are standardized to mean = 0 and SD = 1 within each study

We computed a smoking methylation polyepigenetic score (SmPEGS) for tobacco exposure based on the 2623 CpG probes identified by Joehanes et al.^2,11^ in their epigenome-wide meta-analysis of current vs. never smoking (Supplementary Table S1). We calculated SmPEGS values by multiplying the % methylation measured at a CpG site with the effect-size estimated for that CpG by Joehanes et al.^2^ and then computing the average across the set of CpGs. The resulting SmPEGSs were differentially distributed in never-, former- and current-smokers in the Dunedin and E-Risk cohorts. In the Dunedin cohort, as compared to never-smokers (*n* = 405), former-smokers (*n* = 233) had SmPEGSs on average 0.45 SD units higher [95%CI 0.33–0.58] and current-smokers (*n* = 165) had SmPEGSs on average 1.65 SD units higher [95%CI 1.51–1.80] (*p* < 0.001 for both comparisons; Fig. 1a). In the E-Risk cohort, as compared to never-smokers (*n* = 1203), former-smokers (*n* = 57) had SmPEGSs on average 0.21 SD units higher [95% CI 0.02–0.40] and current-smokers (*n* = 374) had SmPEGSs on average 1.00 SD units higher [95% CI 0.87–1.13] (*p* = 0.03 for former-smokers; *p* < 0.001 for current-smokers; Fig. 1b). Supplementary Fig. S2 shows SmPEGS successfully discriminated never- from both current- and ever-smokers in both Dunedin and E-Risk (AUC range from 0.77–0.93).

#### Methylation polygenic scores tracked upwards with cumulative cigarette consumption

Dunedin Study members have been interviewed repeatedly about their smoking behavior since age 15 years^17^. We used these prospective data to calculate the number of pack-years each Study member had smoked through the most recent interview at age-38 years. At age-38 years, for Dunedin Study members who had ever smoked (*n* = 397), pack-years ranged from 0.05–38.75 (mean = 11.71, SD = 8.65). Those who had smoked more pack-years had higher SmPEGS; each additional pack-year smoked was associated with a 0.07 SD unit higher SmPEGS [95% CI 0.06–0.08] (*p* < 0.001) (Fig. 1c).

Next, we turned to the 18-year-olds in the E-Risk sample. At age-18 years, for E-Risk Study members who had ever smoked (*n* = 431), pack-years ranged from 0.05–14.85 (mean = 2.04 SD = 1.76). Those who had smoked more pack-years had higher SmPEGS; each additional pack-year smoked was associated with a 0.1 SD unit higher SmPEGS [95% CI 0.05–0.15] (*p* < 0.001), similar to the 0.07-unit increase estimated in the Dunedin data (Fig. 1d).

### B. Tobacco smoking has independent causal effects on DNA methylation

#### Smoking and DNA methylation polygenic scores are heritable, but their association is not fully accounted for by genetic variation

Using the twin design of the E-Risk Study^18^, we tested genetic influences on smoking behavior. Consistent with previous research^19^, we find a substantial proportion of variation in pack-years smoked is under genetic influence (rMZ = 0.69, rDZ = 0.45; additive genetic variance = 48.5% [95%CI 35.5–61.8%]; shared environmental variance = 16.0% [95%CI 4.1–27.3%]; non-shared environmental variance = 35.5% [95% CI 31.8–39.7%]; Supplementary materials). In parallel, we found that a substantial proportion of variation in SmPEGS is under genetic influence (rMZ = 0.87, rDZ = 0.52; additive genetic variance = 70.9% [95% CI 60.4–82.6%]; shared environmental variance = 16.0% [95% CI 4.3–26.5%]; non-shared environmental variance = 13.1% [95% CI 11.7–14.6%]).

To test if genetic and shared environmental factors might confound associations between tobacco exposure and SmPEGS values, we studied differences between twins. Specifically, we parsed the effect of pack-years smoked on SmPEGS into between-twin and within-twin-pair effects (Supplementary materials). Within-twin-pair differences in pack-years among both DZ and MZ twins were significantly associated with differences in SmPEGS, such that the co-twin who smoked more had higher SmPEGS (*b* = 0.18, [95% CI 0.10–0.25] *p* < 0.001). We found a similar pattern when the analysis was repeated using only MZ twins (*b* = 0.09, [95% CI 0.02–0.16] *p* = 0.01), indicating the association could not be fully explained by shared family-wide environmental or genetic factors.

#### Study members who smoked additional pack-years between ages 26 and 38 experienced increase in their methylation polygenic scores

Dunedin Study members provided DNA for analysis of methylation at two time points, ages 26 and 38. To isolate the effect of *accumulating* tobacco exposure on *changes* in the methylome, we utilized the repeated-measures of tobacco exposure and SmPEGS in the Dunedin Study. For Study members who accumulated pack-years between ages 26 and 38 (*n* = 280), we regressed change in SmPEGS on the number of additional pack-years smoked. Compared to their age-26 baseline, each additional pack-year smoked between ages 26 and 38 years was associated with a 0.05 SD unit increase in SmPEGS [95% CI 0.03–0.07] (Fig. 1e). Accumulating tobacco exposure is reflected in corresponding SmPEGS change.

#### Dunedin Study members who quit smoking between methylation measurements at ages 26 and 38 experienced a relative decline in their methylation polygenic scores

**Fig. 2:**
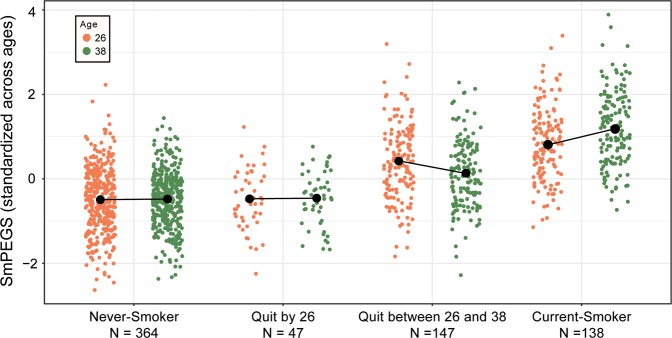
Distribution of SmPEGS in the Dunedin Study at two time points across 12 years as a function of smoking history at age-38. The figure shows Smoking methylation Polygenic Score (SmPEGS) were reduced for those who ceased to smoke between the two DNA methylation assessments at ages 26 and 38. Black points represent means within each group connected by lines across the two assessment phases

Figure 2 describes SmPEGS changes over time in four groups; individuals who have never smoked, those who quit smoking by age-26, those who quit between the two assessments at ages 26 and 38, and those who were still smoking at age-38. For individuals who had never smoked or quit smoking by age-26, SmPEGS stayed relatively static between ages 26 and 38 (mean SD unit change = −0.04 [95% CI −0.1−0.01] for never-smokers, and −0.04 [95% CI −0.19–0.11] for those who quit by age-26). Those individuals still smoking at age-38 experienced an increase in their SmPEGS compared to age-26 (mean SD unit change = 0.49 [95% CI 0.36–0.62]). However, those who ceased smoking between ages 26 and 38 experienced a relative decline in their SmPEGS over the same period (mean SD unit change = −0.25 [95% CI −0.36 to −0.14]); compared to the never-smokers, SmPEGS declined 0.21 SD units [95% CI 0.09–0.32]) over this period (Fig. 2). Ceasing tobacco use is reflected in corresponding SmPEGS recovery.

#### Dunedin Study members who are nicotine dependent have higher methylation polygenic scores than non-dependent smokers, and these scores are higher even after taking into account density of tobacco consumption

In addition to the analysis of changes in SmPEGS related to changes in smoking behavior over time, we conducted analysis of changes in SmPEGS related to changes in nicotine dependence using the Fagerstrom Test of Nicotine Dependence (FTND) at ages 26 and 38^17,20^. This instrument is designed to test dependence on nicotine by assessing, for example, criteria related to frequent urges, valuing smoking most after abstinence, and smoking density. Within those reporting current smoking at age-26, nicotine-dependent Study members had SmPEGS 0.43 SD units [95% CI 0.20–0.65] higher than those not meeting criteria for nicotine dependence. Similarly, at age-38, nicotine-dependent Study Members had SmPEGS 0.61 SD units [95% CI 0.34–0.88] higher than non-dependent Study Members. For individuals who were never dependent, SmPEGS stayed relatively stable between ages 26 and 38 (mean SD unit change = −0.04 [95% CI −0.12–0.05]). Those individuals who were dependent only at age-26 experienced a slight decline in their SmPEGS between ages 26 and 38 (mean SD unit change = –0.12 [95% CI −0.36–0.11]). Those individuals who were dependent at either age-38 only, or at both ages, experienced relative increases in their SmPEGS between ages 26 and 38 (mean SD unit change = 0.58 [95% CI 0.25–0.91] for age-38 only dependent individuals, and 0.60 [95% CI 0.33–0.87] for those who are dependent at both ages). Compared to smokers who do not meet diagnostic criteria at either age, SmPEGS increased 0.62 SD units [95% CI 0.30–0.93] and 0.63 SD units [95% CI 0.40–0.87] in these two groups, respectively (Supplementary Fig. S3). Because diagnosis of nicotine dependence can rely heavily on density of smoking (dependent smokers have on average 8.73 [95% CI 6.96–10.50] more pack-years by age-38 than non-dependent smokers), and because we have demonstrated SmPEGS increase with cumulative tobacco consumption, we further tested whether these associations with FTND were accounted for by accelerated accumulation of tobacco between the two ages. Compared to smokers who do not meet diagnostic criteria at either age, SmPEGS increased 0.37 SD units [95% CI 0.02–0.73] and 0.35 SD units [95% CI 0.05–0.66] for individuals who were dependent at either age-38 only, or at both ages, respectively, when change in number of pack-years between age-26 and 38 was included as a covariate in the analysis. Diagnosis of nicotine dependence predicts inter-age increases in SmPEGS in these two groups even after controlling for accumulation in pack-years between 26 and 38, suggesting the SmPEGS indexes physical aspects of smoking behavior above and beyond volume of cigarette consumption.

#### DNA methylation differences identified in epigenome-wide association studies of smoking relate to gene expression differences in both smokers and non-smokers

DNA methylation has been shown to play a role in mediating gene expression, both directly and indirectly^21–24^. We tested whether the SmPGS, a composite measure of 2623 smoking-related DNA methylation sites, predicts differences in global gene expression by conducting transcriptome-wide analysis of the SmPGS in Dunedin study participants at age-38. This analysis identified 143 differentially expressed probesets within 98 genes (Supplementary Table S2.). Pathway analysis revealed that networks related to cellular movement, immune cell trafficking, hematological system development and function, cell-mediated immune response, and inflammatory response were the most significantly enriched for these genes (Supplementary Table S3.).

We then asked whether individual DNA methylation probes were responsible for these associations by deconstructing the SmPGS. We conducted integrative genomic analysis by testing correlations between methylation levels at the 2623 DNA methylation probes included in the SmPGS and expression levels of the 143 gene expression probesets. To investigate any possible underlying biological relationship between these correlated methylation probes and expression probesets, we conducted this analysis separately in two groups of individuals: those who currently smoke and those who have never smoked. This rationale deems that if a relationship between DNA methylation and gene expression is not dependent solely on shared influence of smoking, then it should be observable both in individuals who have never smoked as well as in those who have. If the relationship is driven by smoking, then it would be observable in just one of these groups. Of the 375,089 correlations tested, 108 were significant after Bonferroni correction (*p* < 1.33 × 10^-7^) in both non-smokers and smokers, representing 47 unique DNA methylation probes and 22 unique gene expression probesets in 14 genes (Supplementary Table S4.).

**Fig. 3:**
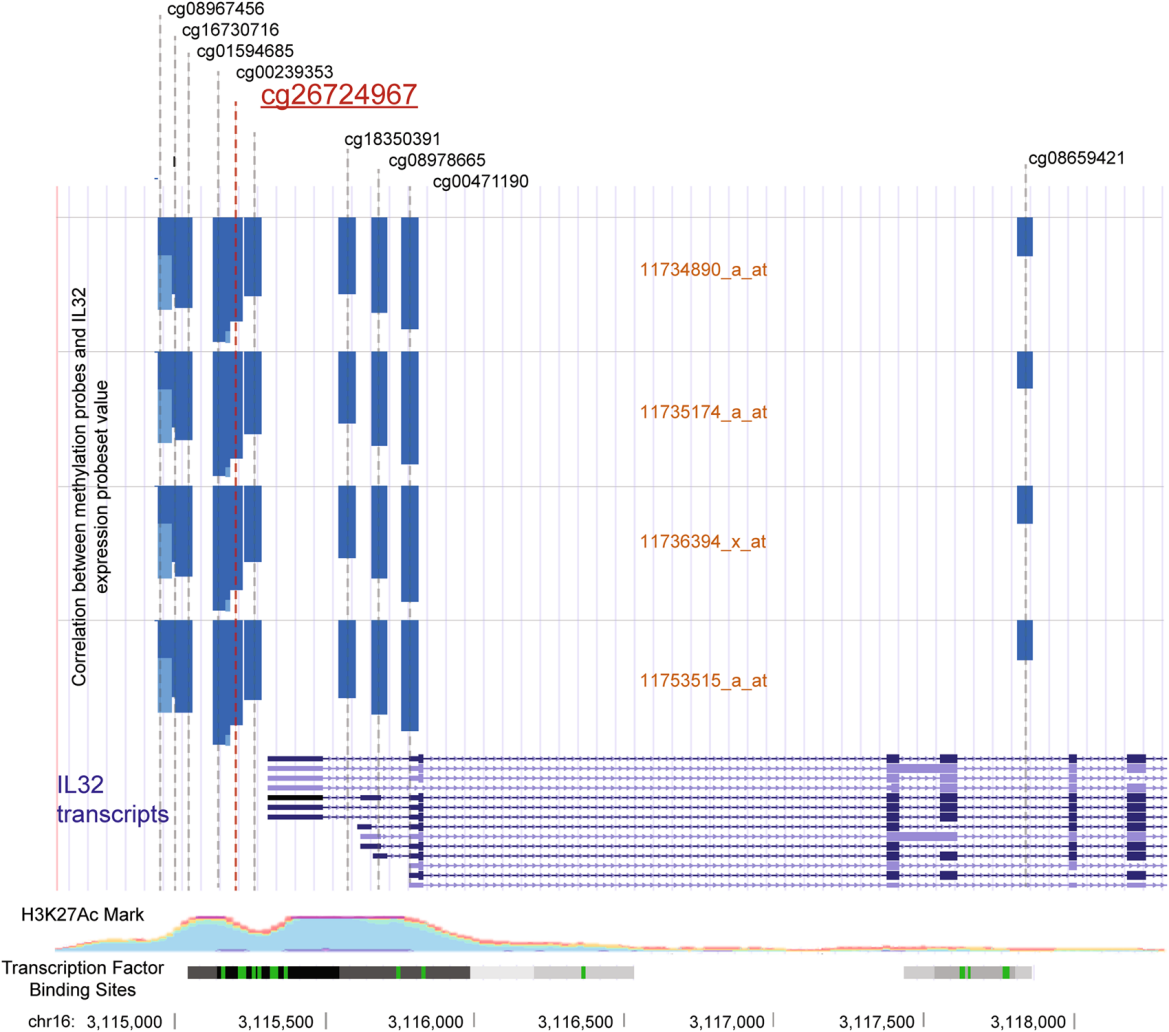
Diagrammatic representation of the region surrounding the transcription start site of IL32. DNA methylation levels of probe cg26724967 (a constituent of the SmPGS) are negatively correlated with *IL32* gene expression levels in the Dunedin Study. Shown are the locations of all 450K array DNA methylation probes in this region (based on genome assembly hg19), with cg26724967 highlighted in red. Each of the four following rows represent the four available probesets for *IL32* on the Affymetrix PrimeView array, and the blue bars indicate the magnitude of the negative correlation between each probeset and DNA methylation probe in the Dunedin Study. All DNA methylation probes in this region show high negative correlations with *IL32* expression, suggesting a role in *IL32* gene regulation. For illustrative purposes, patterns of H3K27Ac marks (indicative of enhancer regions) and transcription factor binding site (TFBS) data from the ENCODE project are shown at the bottom of the plot; peaks of histone modification and multiple ChIP-seq identified TFBS in this location support the hypothesis that the region is important for gene regulation

Most of the correlations between DNA methylation probes and expression probesets identified above describe *trans* relationships; only one of the 47 DNA methylation probes was *cis* (defined as 250 Kb up- or down-stream of the start of a gene) to a correlated probeset, cg26724967 located 124 bp upstream of the start of *IL32*. This DNA methylation probe is negatively correlated with four probesets indexing *IL32* gene expression (Fig. 3). IL32 is an inflammatory cytokine that has been previously associated with COPD and smoking-related damage to the lungs^25,26^. *Cis* relationships between DNA methylation and *IL32* expression have been previously observed in smokers^2^, but it is not clear that the relationship is independent of smoking; here we document *cis* relationships in non-smokers, which suggests the potential for a causal biological effect of proximal DNA methylation on *IL32* expression independent of smoking. IL32 is an excellent candidate for understanding the biological impact of DNA methylation on disease risk.

As a sensitivity analysis, we entertained the possibility that methylation-expression correlations among non-smokers arise because of passive exposure to second-hand smoking that exerts similar effects to the direct consumption of tobacco products. We repeated the correlation analyses in the never-smoker group while controlling for self-reports of exposure to second-hand smoke (for example, in the home or workplace). The magnitude and direction of correlations between DNA methylation probes and gene expression probesets were comparable to the non-adjusted correlation statistics (spearman’s *rho* = 0.99, *p* < 0.001, Supplementary Table S4), suggesting the observed associations reflect biological relationships not driven by underlying passive tobacco consumption.

### C: Methylation polygenic score changes between 26 and 38 years track smoking-associated damage to lungs and gums

Our longitudinal analysis in the Dunedin cohort supports the hypothesis that SmPEGS tracks the accumulation of tobacco exposure and SmPEGS becomes attenuated over time among those who quit smoking. An important question, therefore, is whether SmPEGS reflects processes of smoking-associated physiological damage and recovery.

We considered damage to two systems proximate to tobacco exposure: the lungs and the gums. Our analysis of lung and gum damage used longitudinal data collected at the same time as DNA methylation. Lung function was measured at age-26 and 38 years using the carbon monoxide transfer coefficient DLco/VA (in mL/min/mmHg/L), an index of the efficiency of alveolar transfer of carbon monoxide; lower values index worse lung function. Gum health was measured at ages 26 and 38 years as periodontal attachment loss in mm; higher values index worse gum health.

#### Lung function

**Fig. 4:**
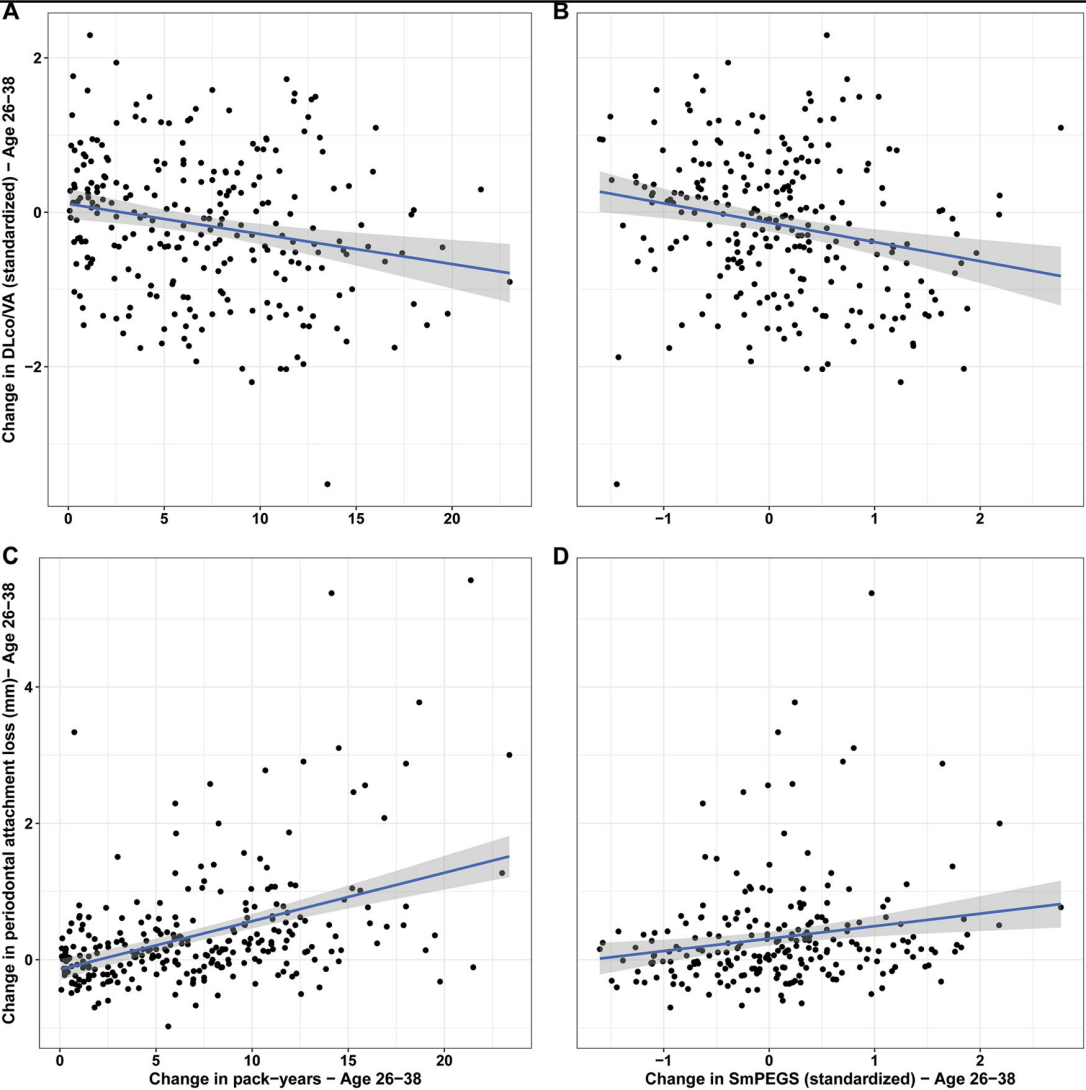
Association between change in pack-years smoked or SmPEGS and change in smoking-associated damage to lungs and gums between ages 26 and 38 in the Dunedin Study. (**a**) and (**c**) show change in pack-years versus change in lung function and peridontal attachement loss, repectively. (**b**) and (**d**) show change in SmPEGS and change in lung function and periodontal attachment loss, respectively. The analysis is restricted to study members who smoked between ages 26 and 38 years

Study members who smoked at ages 26 and 38 years suffered damage to their lungs. Current-smokers at age-26 had DLco/VA measures 0.28 units lower than never-smokers, and at age-38 current-smokers had measures 0.83 units lower than never-smokers. Longitudinally, we find that within-individual changes in pack-years accumulation tracked with within-individual changes in lung function. Among those who had ever smoked, each additional pack-year smoked was associated with a 0.03-SD unit decline in DLco/VA [95%CI 0.01–0.05] (Fig. 4a). In parallel, we tested whether study members’ SmPEGS was associated with lung damage. Each additional SmPEGS SD unit increase was associated with a 0.23-SD unit decline in DLco/VA [95% CI 0.10–0.36] (Fig. 4b).

#### Gum health

Study members who smoked at ages 26 and 38 years suffered damage to their gums. Current-smokers at age-26 had 0.13 mm more periodontal attachment loss than never-smokers, and at age-38 current-smokers had 0.77 mm more periodontal attachment loss than never-smokers. Longitudinally, we find that within-individual changes in pack-years accumulation tracked with within-individual changes in periodontal attachment loss. Among those who had ever smoked, each additional pack-year smoked was associated with 0.07 mm of additional periodontal attachment loss [95% CI 0.05–0.08] (Fig. 4c). In parallel, we tested whether study members’ SmPEGS was associated with gum damage. Each additional SmPEGS SD unit increase was associated with 0.17 millimeters of additional periodontal attachment loss [95% CI 0.06–0.28] (Fig. 4d).

These findings suggest that not only does the SmPEGS track smoking, but also smoking-related damage to biological systems. This damage is detectable external to the tissue of clinical interest, namely the lungs and gums. The SmPEGS, measurable in a blood sample, may serve as a suitable proxy when invasive or complicated testing are not feasible.

### D: Cigarette smoking confounds associations between psychosocial risk factors and DNA methylation

Adverse experiences in childhood are associated with increased morbidity and early mortality^16^. Epigenetic modifications are thought to play a role in this apparent “biological embedding” of risk^27–29^. However, adverse childhood experiences (ACEs) are also associated with smoking behavior^30^, making it difficult to tease apart epigenetic associations with early stress from the effects of smoking on the epigenome^31^.

We conducted EWAS of reports of ACEs in the Dunedin and E-Risk studies. In both cohorts, we measured ACEs as the sum of exposure to ten types of childhood experiences. ACEs predicted differential DNA methylation at ten probes in the Dunedin Study and seven in the E-Risk Study. However, each one of these probes was associated with smoking in our Dunedin and E-Risk studies, and in the study by Joehanes et al.^2^. After statistical control for number of pack-years smoked, no probes remained statistically significantly associated with ACEs in the Dunedin Study and only one probe (cg25949550) remained significant in the E-Risk Study (Supplementary Table S5).

**Fig. 5:**
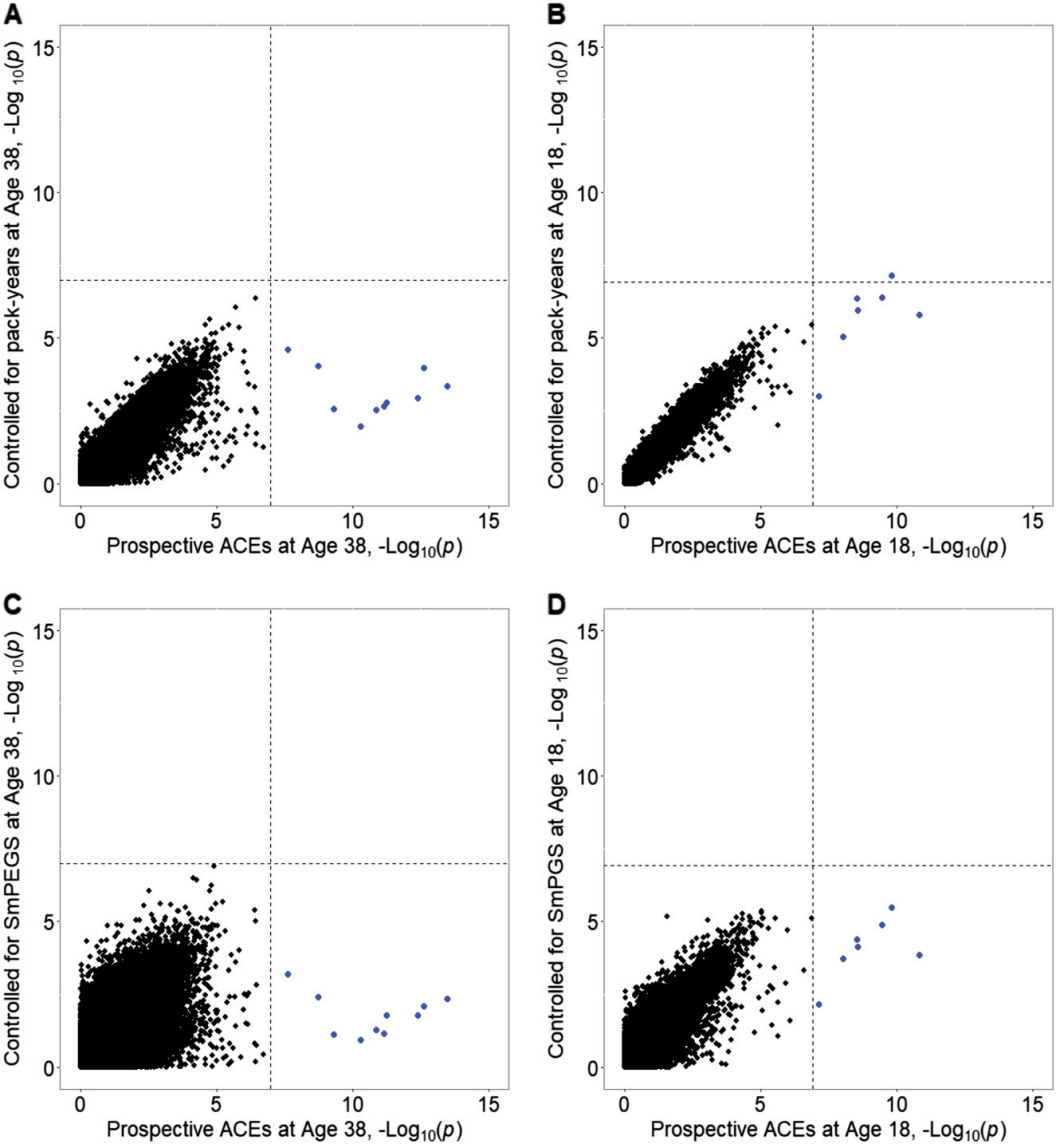
Cigarette smoking confounds associations between psychosocial risk factors and DNA methylation. **a**, **b** show the scatterplot of the –Log_10_(*p*) values from an epigenome-wide association studies (EWAS) of adverse childhood experiences (ACEs; *x*-axis) plotted against –Log_10_(*p*) from an EWAS of ACEs controlling for pack-years smoked at age-38 in the Dunedin Study (**a**) and age-18 in the E-Risk Study (**b**). **c**, **d** show the same scatterplot but substituting SmPEGS at age-38 in Dunedin (**c**) and age-18 in E-Risk (**d**) for pack-years smoked. Dashed lines represent the genome-wide significant cutoff levels. Blue points represent probes significant in the EWAS of ACEs. Had EWAS hits for ACEs remained significant after controlling for smoking (**a**, **b**) or for Smoking methylation Polygenic Score (SmPEGS) (**c**, **d**), the blue points would appear in the upper right-hand quadrant. In both cohorts, controlling for smoking or using the SmPEGS attenuates the association between ACEs and DNA methylation to non-significance; hence the blue points appear in the lower right-hand quadrant

We further tested if including the SmPEGS as a covariate would yield a similar pattern of attenuation to that observed when including smoking history as a covariate. We found that comparable statistical control for smoking could be achieved using the SmPEGS (Fig. 5). Statistical control for smoking history is vital when conducting epigenetic analyses of ACEs, and if a study lacked observed smoking history data, the SmPEGS could serve as a suitable proxy control.

## Discussion

We demonstrated that DNA methylation patterns in whole blood are associated with tobacco smoking, and that composite scores comprising specific DNA methylation sites (SmPEGS) can be utilized to index smoking behavior. SmPEGS are higher in smokers than non-smokers, are observable in both middle-aged individuals (Dunedin Study members) and young adults (E-Risk Study members), increase with additional tobacco consumption, and subside upon smoking cessation. In parallel with smoking, changes in SmPEGS over time index changes in lung function and gum health. Lastly, tobacco smoking confounds DNA methylation studies of psychosocial risk, and this can be accounted for by introducing either observed smoking history or SmPEGS as a statistical control.

There are a number of implications of SmPEGS. First, analysis of twins in the E-Risk Study shows that, while there is substantial familiality to SmPEGS, the association with smoking cannot be fully accounted for by either genetics or shared environmental effects. This is in line with several smaller twin studies that have focused on DNA methylation at individual probe sites^32,33^, and other research that demonstrates that while methylation Quantitative Trait Loci (mQTLs) for smoking-related probes exist, a substantial amount are independent of genetic correlates^34^. These observations combined suggest that smoking might elicit effects on DNA methylation independent of background risk associated with an individual’s genetic or environmental propensity for smoking.

Second, changes in SmPEGS parallel smoking behavior changes; Dunedin Study members who continued to smoke between age-26 and age-38 saw a corresponding increase in their SmPEGS between assessments, while those who ceased smoking over the same period saw a relative decline in their SmPEGS. Dynamic responses of DNA methylation levels to changes in smoking behavior are of particular significance to public health; if DNA methylation changes represent clinically relevant indicators of damage arising from tobacco use, then their reversal after ceasing tobacco use suggests the potential for biological recovery. Previous studies have demonstrated that CpG site-specific DNA methylation has the potential for long-term recovery via time-since-quit models of analysis^2,5^, although the results are not consistent^4,8,35,36^. Here, using within-person changes in smoking behavior mapped to within-person changes in SmPEGs, we show that there is potential for recovery at the aggregate level.

Third, a number of DNA methylation sites that comprise SmPEGS predict corresponding changes in gene expression. These differentially expressed genes are found in pathways relating to inflammation, immune response, and cellular trafficking, indicating smoking-induced DNA methylation differences might exert an effect via inflammatory pathways. Moreover, a proportion of these correlations are observed in those who do not smoke, suggesting these DNA methylation-gene expression relationships are not artefacts of smoking behavior. That these DNA methylation sites were initially identified through differential methylation patterns in response to smoking gives rise to possible routes by which smoking exerts biological damage at the molecular level. Of particular interest is the DNA methylation probe cg26724967, negatively correlated in *cis* to *IL32* expression. IL32 is a relatively recently discovered cytokine, and previous research has shown that *IL32* mRNA and protein levels in serum and lung tissue are higher in smokers with chronic pulmonary obstructive disease (COPD) than non-smoking COPD patients, non-COPD smokers, and healthy controls^25,26^. IL32 is an excellent candidate biomarker for smoking-induced damage to health, and warrants further investigation.

Fourth, changes in SmPEGS index changes in lung function and gum health over time; these changes are comparable to those predicted by changes in amount of cigarettes smoked over the same period. This suggests SmPEGS can be utilized to index damage to distal biological systems (in this case, the lungs and gums) without necessitating collection of said tissue. Evidence exists that some of these smoking-sensitive DNA methylation sites can also be identified in lung tissue^37,38^, and further research on the link between DNA methylation in blood and damage to distal biological systems could uncover novel biomarkers of health^39^.

The final implication concerns future study design^40,41^. Epigenome-wide association studies strive to remove effects of confounding covariates^42^ when the confounding factor is robustly associated with (a) the exposure/phenotype under test, and with (b) differential DNA methylation. These two points are relevant in studies of psychosocial stress, which are vigorously interrogating how “stress gets under the skin”. A difficulty is that individuals who have higher levels of stress smoke more^30^, and smoking itself elicits differential effects on the epigenome. Here, we show that in the case of adverse childhood experiences (ACEs), smoking confounds tests of the association with DNA methylation. When testing associations between DNA methylation and a phenotype known to be also associated with smoking (e.g., schizophrenia, alcoholism, hypertension^43,44^), statistical control for smoking behavior should be introduced. Where a subject’s smoking history is undetermined or potentially unreliable^7^, SmPEGS can be utilized successfully in its place. Some researchers have started to use polyepigenetic scores to index smoking, but they are developing these scores in idiosyncratic and sample specific ways. This is likely to impede the systematic accumulation of knowledge. Thus, here we have developed a standard methodology for creating polyepigenetic scores. Importantly, this framework of score construction can be extended to any measure associated with both an exposure and DNA methylation so as to generate proxy controls for confounders where necessary.

There are some caveats. First, we took a conservative approach to constructing SmPEGS by utilizing only the 2623 probes that reached genome-wide significance (*p* < 1 × 10-07) in Joehanes et al.^2^. While this approach of restricting candidate markers to those with association *p*-value less than a defined threshold is common in the polygenic scoring community^45,46^, it is possible we omitted some biologically relevant variation in smoking-related DNA methylation not captured by this list of probes. However, as shown in Supplementary Materials (Supplementary Table S6), we also constructed a score utilizing all 18,760 probes that reached False Discovery Rate (FDR)-level significance in Joehanes et al.^2^. This score predicted smoking no better than the SmPEGS. In contrast to the polyepigenetic approach, a primary focus of previous biomarker development has been the single CpG probe cg05575921, located within the gene *AHRR*. Indeed, consistent with prior reports, methylation beta levels of this one probe do an excellent job of discriminating never-, former-, and current-smokers and is associated with cumulative smoking exposure in our data^6,7,47^ (Supplementary Table S6). Reported associations between cg05575921 and smoking are not always consistent; for example, evidence of rebound to non-smoking levels after quitting is conflicting^35,36,48^, and evidence of association with dependence-associated behavior is lacking^6^. To this end, it is possible that a polyepigenetic approach to assessing smoking dynamics could better capture divergent underlying biological correlates of smoking, such as nicotine craving, that a single DNA methylation signal does not. Second, we constructed SmPEGS in blood DNA; we are unable to verify whether SmPEGS are generalizable across different tissue types. It is likely that the tissue specificity of DNA methylation levels varies across the range of probes used to construct the SmPEGS^49^. We encourage the validation of cross-tissue applicability when substrates other than blood are used. That said, given that most population-based assessments of DNA methylation are made using DNA from blood, the algorithm for construction of SmPEGS described herein has broad-scale utility for the majority of researchers. Third, we borrowed the idea of a polyepigenetic score from the methods employed by the GWAS community, and we assume that the effects are additive. As in this cognate area^46^, this assumption will require validation and interrogation.

In conclusion, we describe the construction of a DNA methylation composite score that indexes smoking behavior, predicts damage to biological systems known to be affected by smoking, is correlated with changes in gene expression, and can be utilized as a proxy smoking-history measure in situations where observational smoking data are unavailable.

## Supplementary information

Supplementary Material.

Supplementary Table S1.

Supplementary Table S2.

Supplementary Table S3.

Supplementary Table S4.

Supplementary Table S5.

Supplementary Table S6.
