# Supplementary material for: Establishing a generalized polyepigenetic biomarker for tobacco smoking
Source: Transl Psychiatry. 2019 Feb 15;9:92. doi: 10.1038/s41398-019-0430-9 (PMC6377665; doi:10.1038/s41398-019-0430-9)

**SUPPLEMENTARY INFORMATION TO:**

**Establishing a Generalized Polyepigenetic Biomarker for Tobacco Smoking.**

Sugden *et al*.

This Supplement contains additional details about the samples, genome-wide quantification of DNA methylation and gene expression, and measurement of smoking history, health measures and Adverse Childhood Experiences. In addition, it contains additional figures and tables to accompany statistical analyses reported in the Main Article. The analysis plan for this paper was posted in advance (http://www.moffittcaspi.com).

**Dunedin Longitudinal Study**

Sample description. Participants were members of the Dunedin Multidisciplinary Health and Development Study, a longitudinal investigation of health and behavior in a representative birth cohort (1). Study members (n = 1,037; 91% of eligible births; 52% male) were all individuals born between April 1972 and March 1973 in Dunedin, New Zealand, who were eligible for the longitudinal study based on residence in the province at 3 years of age and who participated in the first follow-up assessment at 3 years of age. The cohort represented the full range of socioeconomic status on NZ’s South Island. On adult health, the cohort matches the NZ National Health and Nutrition Survey (e.g., BMI, smoking, GP visits(1)). Cohort members are primarily white; approximately 7% self-identify as having partial non-Caucasian ancestry, matching the South Island. Assessments were carried out at birth and at ages 3, 5, 7, 9, 11, 13, 15, 18, 21, 26, 32, and 38 years, when 95% of the 1,007 study members still alive took part. The Otago Ethics Committee approved each phase of the study and informed consent was obtained from all study members.

Genome-wide quantification of DNA methylation. Our epigenetic study used DNA from a single tissue: blood. Whole blood was collected in 10mL K_2_EDTA tubes from 93 % (N=836) of the non-Maori participants at age 26, and 90% (N=857) at age 38. DNA was extracted from the buffy coat using standard procedures (2, 3). Study members who did not provide blood provided buccal swabs, but these were not included in our methylation analysis to avoid tissue-source confounds.

At age 26 we assayed 833 blood samples (out of 836); 3 samples were not useable (e.g., due to low DNA concentration). At age 38 we assayed 835 blood samples (out of 857); 22 samples were not useable. ~500ng of DNA from each sample was treated with sodium bisulfite using the EZ-96 DNA Methylation kit (Zymo Research, CA, USA). DNA methylation was quantified using the Illumina Infinium HumanMethylation450 BeadChip (“Illumina 450K array”) run on an Illumina iScan System (Illumina, CA, USA) at the Molecular Genomics Core at the Duke Molecular Physiology Institute. Age-26 and -38 DNA samples from the same individual were processed in adjacent positions to minimize batch effects.

Data were processed and normalized using the ‘methylumi’ (v2.14.0) Bioconductor package from the R statistical programming environment, and subjected to quality control (QC) analyses. Samples were removed if the average detection p-value was >= 0.001. To confirm genetic identity of the DNA samples, we assessed genotype concordance between SNP probes on the 450K array and data generated using Illumina OmniExpress12v1.1 genotyping BeadChips. Principal components analysis was performed on the full, normalized dataset and the first two components plotted (4). Samples formed two major clusters separating on the 1st component, which corresponded to recorded sex. This was used to confirm sex assignment. Samples from 818 age-26 and 819 age-38 participants passed our QC pipeline; after exclusion of participants due to pregnancy, DNA methylation data were available for 776 age-26 and 804 age-38 participants, and at both ages for 697 participants. **Supplementary Figure S1** is a flow chart describing QC steps and sample Ns at each stage.

Gene Expression. Expression data were generated from whole-blood RNA using the Affymetrix PrimeView Human Gene Chip (Affymetrix, CA, USA).Briefly, these arrays simultaneously interrogate more than 38,000 gene transcripts across the entire genome. Whole-blood RNA samples collected via PaxGene Blood RNA tubes (Qiagen, CA, USA) at age 38 were assayed. Samples were arranged into batches of 60. Array analysis was performed by the Duke University Genomic and Computational Biology Microarray Core Facility using the Affymetrix GeneChip system (Affymetrix). Prior to hybridization, Total RNA were assessed for quality with Agilent 2100 Bioanalyzer G2939A (Agilent Technologies, Santa Clara, CA)) and Nanodrop 8000 spectrophotometer (Thermo Scientific/Nanodrop, Wilmington, DE). Samples with RIN ≥ 6 were then subject to globin mRNA depletion using the GLOBINClear –human kit (Ambion, Thermo Fisher Scientific, MA, USA). RNA samples from 843 individuals were assayed. Data quality control and RMA normalization was carried out using the ‘affy’ Bioconductor package in the R statistical programming environment. After QC, expression data was available for 836 individuals.

To permit control for technical variation, we used the following microarray-based quality metrics as covariates in individual-level analyses following the approach described by Peters *et al*. (5): mean of positive match probesets, mean of positive control probesets, standard deviation of positive control probesets, mean of negative control probesets, standard deviation of negative control probesets, mean of all probesets, standard deviation of all probesets, and relative log expression mean of all probesets, along with sex, array batch and RIN. To control for cell type composition, we used as covariates white cell-type counts measured using flow cytometry (Sysmex Corporation, Japan) in whole blood samples taken concurrently with the RNA sample.

**Environmental Risk (E-Risk) Longitudinal Twin Study**

Participants were members of E-Risk, which tracks the development of a 1994-95 birth cohort of 2,232 British children (6). Briefly, the E-Risk sample was constructed in 1999-2000, when 1,116 families (93% of those eligible) with same-sex 5-year-old twins participated in home-visit assessments. This sample comprised 56% monozygotic (MZ) and 44% dizygotic (DZ) twin pairs; sex was evenly distributed within zygosity (49% male). The study sample represents the full range of socioeconomic conditions in Great Britain, as reflected in the families’ distribution on a neighborhood-level socioeconomic index (ACORN [A Classification of Residential Neighbourhoods], developed by CACI Inc. for commercial use): 25.6% of E-Risk families live in “wealthy achiever” neighborhoods compared to 25.3% nationwide; 5.3% vs. 11.6% live in “urban prosperity” neighborhoods; 29.6% vs. 26.9% in “comfortably off” neighborhoods; 13.4% vs. 13.9% in “moderate means” neighborhoods; and 26.1% vs. 20.7% in “hard-pressed” neighborhoods. E-Risk underrepresents “urban prosperity” neighborhoods because such households are often childless.

Home visits were conducted when participants were aged 5, 7, 10, 12 and most recently, 18 years (93% participation). The Joint South London and Maudsley and the Institute of Psychiatry Research Ethics Committee approved each phase of the study. Parents gave informed written consent and twins gave written assent between 5-12 years and then informed written consent at age 18.

At age 18, 2,066 participants were assessed, each twin by a different interviewer. The average age at the time of assessment was 18.4 years (SD = 0.36); all interviews were conducted after the 18th birthday.

Genome-wide quantification of DNA methylation. Our epigenetic study used DNA from a single tissue: blood. At age 18, whole blood was collected from 82% (N=1700) of the participants in 10mL K_2_EDTA tubes. DNA was extracted from the buffy coat using a Flexigene DNA extraction kit (Qiagen, Hilden, Germany) following manufacturer’s instructions. Study members who did not provide blood provided buccal swabs, but these were not included in our methylation analysis to avoid tissue-source confounds.

We assayed 1669 blood samples (out of 1700); 31 samples were not useable (e.g., due to low DNA concentration). ~500ng of DNA from each sample was treated with sodium bisulfite using the EZ-96 DNA Methylation kit (Zymo Research, CA, USA). DNA methylation was quantified using the Illumina Infinium HumanMethylation450 BeadChip (“Illumina 450K array”) run on an Illumina iScan System (Illumina, CA, USA) by the Complex Disease Epigenetics Group at the University of Exeter Medical School. Twin pairs were randomly assigned to bisulfite-conversion plates and Illumina 450K arrays, with siblings processed in adjacent positions to minimize batch effects. Fully methylated control samples (CpG Methylated HeLa Genomic DNA; New England BioLabs, MA, USA) were included in a random position on each plate; the distinct DNA methylation profile of this sample enabled us to confirm the experiment was successful and to ensure there were no plate mix-ups or rotations.

Data were imported using the methylumIDAT function in ‘methylumi’ (7), and subjected to quality control analyses. First, we excluded all samples with median methylated (‘M’) and unmethylated (‘U’) intensities <2500. Second, using the ten control probes included on the 450K array, we examined the efficiency of the sodium bisulfite conversion reaction; samples were excluded if their “conversion score” was <80. Third, multidimensional scaling was performed for DNA methylation probes on each of the sex chromosomes and compared to the reported gender. Fourth, to confirm genetic identity of the DNA samples, we assessed genotype concordance between SNP probes on the 450K array and data generated using Illumina OmniExpress24v1.1 genotyping BeadChips.

Data were processed with the pfilter function from the ‘wateRmelon’ package (8) excluding 0 samples with >1% of sites with a detection p value >0.05, 567 sites with beadcount <3 in 5% of samples and 1448 probes with >1% of samples with detection p value >0.05. The data were normalized with the dasen function from the ‘wateRmelon’ package (8). Prior to any analyses, probes with common (> 5% MAF) SNPs within 10 bp of the single base extension and probes with sequences previously identified as potentially hybridizing to multiple genomic loci were excluded (9, 10), resulting in a final dataset of 430,802 probes. Samples from 1,658 E-Risk twins passed our QC pipeline, including 734 complete twin pairs (58% MZ). **Supplementary Figure S1** is a flow chart describing QC steps and sample Ns at each stage.

**Analysis Variables**

Smoking Methylation Polygenic Score (SmPEGS). We computed a methylation polygenic score (SmPEGS) based on the epigenome-wide association study (EWAS) of smoking status by Joehanes *et al*. (11). We composed our score from the 2,623 CpGs identified as genome-wide significant (i.e. p<1x10^-7^) in analysis of never- vs current smoking. To compute the score, we first matched CpG probe-IDs in each of our datasets with CpG probe-IDs in the meta-analysis results. We next multiplied the probe intensities for these CpGs in our data by the coefficients estimated in the meta-analysis. Data were available on all 2,623 probes In the Dunedin Study and 2,480 probes in the E-Risk Study. Finally, we computed the average of weighted probe intensities across the set of probes. Illumina probe IDs of the included probes can be found in **Supplementary Table S1**.

To permit control for technical variation, we used methylation-array control-probe principal components (4). In the Dunedin Study, 32 PCs were needed to explain 90% of the variance, and in the E-Risk Study 28 PCs were needed. These PCs were used as covariates in all individual-level analyses. To control for cell type composition, we used as covariates white blood cell measures. In the Dunedin Study, white cell-type counts were measured using flow cytometry (Sysmex Corporation, Japan) in whole blood samples taken concurrently with the DNA sample. Observed cell type counts included were monocytes, basophils, neutrophils, eosinophils and lymphocytes. In the E-Risk Study, white cell-type proportions were estimated from the methylation data (12). Estimated cell types included plasma blasts, CD8+CD28-CD45RA- T cells, naïve CD8 T cells, CD4 T cells, natural killer cells, monocytes, granulocytes. To control for ethnicity, we used as covariates the first two principal components computed from analysis of genome-wide SNP data using using PLINK v1.9 (13).

The SmPEGS were generated using the residuals from these equations, standardized to mean = 0 and SD =1 within each of the three separate samples (Dunedin samples at ages 26 and 38 and E-risk samples at age 18). Additionally, for the longitudinal analysis in the Dunedin Study, we standardized SmPEGS across ages 26 and 38 (i.e. standardized to mean = 0 and SD = 1 across both samples). Change scores were calculated as the across-age standardized age-38 SmPEGS – across-age standardized age-26 SmPEGS.

Smoking History. Smoking behavior in Dunedin has been assessed repeatedly between ages 13-38 years, including information about quantity smoked, cessation, and second-hand smoke exposure. In E-Risk, participants were interviewed about their smoking history at age 18 years. For qualitative analysis, individuals reporting no history of smoking were classified as never smokers, individuals reporting any smoking at previous phases but not at the current phase were classified as former smokers, and those reporting current smoking at assessment were classified as current smokers. Specific phenotypes are described in the Results.

Lung health (DLco/VA). Single-breath diffusing capacity for carbon monoxide (DLco/VA) was assessed at ages 26 and 38 in the Dunedin Study according to European Respiratory Society/American Thoracic Society standards using a body plethysmograph (CareFusion, Yorba Linda, CA). DLco/VA is a clinically useful test of lung function which measures the conductance of transfer from gas in the lungs to the red blood cells. DLco/VA measures (in mL/min/mmHg/L) were adjusted for haemoglobin, height, sex, and whether participants had smoked within an hour testing (14).

Gum health (periodontal attachment loss). Examinations were conducted by calibrated dentists registered with the New Zealand Dental Council; three sites (mesiobuccal, buccal, and distolingual) per tooth were examined, and gingival recession (the distance in millimeters from the cementoenamel junction to the gingival margin) and probing depth (the distance from the probe tip to the gingival margin) were recorded using a PCP-2 periodontal probe. The combined attachment loss for each site was computed by summing gingival recession and probing depth (third molars were not included). Periodontal attachment loss is taken as the mean combined attachment loss across the measured sites (15, 16).

Adverse Childhood Experiences (ACEs). The measured ACEs correspond to the 10 subcategories of childhood adversity introduced by the U.S. Centers for Disease Control & Prevention (CDC) Adverse Childhood Experiences Study: three types of abuse (emotional, physical, sexual), five types of household challenges (household partner violence, household substance abuse, mental illness in household, loss of a parent [parental death, separation, or divorce], incarceration of a family member), and two types of neglect (emotional, physical). ACE counts were generated from archival Dunedin Study records collected at biennial assessments from ages 3 to 15 years, as previously described (17). The records included the following: social service contacts; structured notes from assessment staff who interviewed study children and their parents; structured notes from pediatricians and psychometricians who observed mother–child interactions at the research unit; structured notes from nurses who recorded conditions witnessed at home visits; and notes of concern from teachers who were surveyed about the study children’s behavior and performance. ACE counts were generated in the E-Risk Study based on data collected when the Study participants were aged 5, 7, 10 and 12 years, as previously described (18). The records included: interview notes from repeated interviews with the children’s primary caregiver, which were subsequently reviewed and coded by clinicians; structured notes from home visitors who recorded conditions witnessed at home visits; family history assessments; and information about family changes recorded and dated on a Life History Calendar.

**Data analysis**

Data analysis was performed in the R statistical programming environment (version 3.4.2). Data handling was performed using the package ‘dplyr’ (version 0.7.4) and descriptives were generated using package ‘psych’ (version 1.7.8). Plots were produced in R using the packages ‘ggplot2’ (version 2.2.1) and ‘ggpubr’ (version 0.1.6.999).

Association testing. In Dunedin, linear regression was used to test the association between SmPEGS and smoking history, lung function and gum health. In E-Risk, Generalized Estimating Equations (GEE) were used to account for the clustering within families. GEE was performed using the ‘geepack’ package (version 1.2-1) in R. All models included sex as a covariate.

In Dunedin, partial correlations were performed between DNA methylation probe values and gene expression probeset values controlling for passive tobacco smoke exposure using the ‘ppcor’ package in R (version 1.1).

Structural equation modelling. An ACE model was fitted to calculate the proportion of variance in pack years smoked and SmPEGS explained by heritable (A), shared environmental (C) and unshared or unique environmental (E) factors. The model was fitted using structural equation modelling implemented with functions from the ‘OpenMx’ R package (version 2.8.3).

Within-family tests. We used the twin design of the E-Risk Study to account for shared environmental and genetic confounding effects on the association between smoking and SmPEGS. Specifically, a test of the association among twins reared together examines whether smoking and SmPEGS covary solely because of environmental factors shared by the siblings. A test of this association limited to MZ twins reared together, who share 100% of their genes in common, can go one step further and also examines whether smoking and SmPEGS covary because of shared genetic propensity. We parsed the effect of smoking on SmPEGS into between-twin pair effects and within-twin pair effects using a linear regression model with the following specification: *E(Y_ij_)* = β_0_ + β_w_(X*_ij_* - $\bar{X}$*_i_*) + β_B_$\bar{X}$*_i_* , where *i* is used to index twin pairs and j represents individual twins within pairs, so *E(Y_ij_)* and *X_ij_* represent, respectively, the predicted score on SmPEGS and the smoking score for the *j*th twin of the *i*th pair, whereas $\bar{X}$*_i_* represents the mean smoking score for both twins within the *i*th pair. The between-twin-pair regression coefficient (β_B_) estimates whether pairs of twins with higher average pack years tend to have higher SmPEGS at age 18 years. In contrast, the within-twin-pair regression coefficient (β_w_) estimates whether the twin who smokes more than his or her co-twin tends to also have higher SmPEGS than his or her co-twin (19).

Pathway Analysis. Pathway analysis of genes identified in the analysis of DNA methylation – gene expression correlation analysis was performed using Ingenuity Pathway Analysis software (Qiagen).

Genome-wide DNA methylation analysis. Linear regression was used to test the association between smoking and variation at each DNA methylation probe. In E-Risk, Generalized Estimating Equations (GEE) were used to account for the clustering within families. GEE was performed using the ‘geepack’ package in R. To account for technical variation in DNA methylation, PCs generated from the control probes on the Illumina 450K array, observed (Dunedin Study) or estimated (E-Risk Study) cell counts, PCs generated from genome-wide SNP data, and sex were included as covariates.

**References**

1. Poulton R, Moffitt TE, Silva PA. The Dunedin Multidisciplinary Health and Development Study: overview of the first 40 years, with an eye to the future. *Soc Psychiatry Psychiatr Epidemiol*. 2015;**50**(5):679-93.

2. Bowtell DD. Rapid isolation of eukaryotic DNA. *Anal Biochem*. 1987;**162**(2):463-5.

3. Jeanpierre M. A rapid method for the purification of DNA from blood. *Nucleic Acids Res*. 1987;**15**(22):9611.

4. Lehne B, et al. A coherent approach for analysis of the Illumina HumanMethylation450 BeadChip improves data quality and performance in epigenome-wide association studies. *Genome Biol*. 2015;**16**:37.

5. Peters MJ, et al. The transcriptional landscape of age in human peripheral blood. *Nat Commun*. 2015;**6**:8570.

6. Moffitt TE. Teen-aged mothers in contemporary Britain2002 Sep. 727-42 p.

7. Davis S, Du P, Bilke S, Triche JT, Bootwalla M. methylumi: Handle Illumina methylation data. 2017.

8. Pidsley R, CC YW, Volta M, Lunnon K, Mill J, Schalkwyk LC. A data-driven approach to preprocessing Illumina 450K methylation array data. *BMC Genomics*. 2013;**14**:293.

9. Price ME, et al. Additional annotation enhances potential for biologically-relevant analysis of the Illumina Infinium HumanMethylation450 BeadChip array. *Epigenetics Chromatin*. 2013;**6**(1):4.

10. Chen YA, et al. Discovery of cross-reactive probes and polymorphic CpGs in the Illumina Infinium HumanMethylation450 microarray. *Epigenetics*. 2013;**8**(2):203-9.

11. Joehanes R, et al. Epigenetic Signatures of Cigarette Smoking. *Circ Cardiovasc Genet*. 2016;**9**(5):436-47.

12. Houseman EA, et al. DNA methylation arrays as surrogate measures of cell mixture distribution. *BMC Bioinformatics*. 2012;**13**:86.

13. Chang CC, Chow CC, Tellier LC, Vattikuti S, Purcell SM, Lee JJ. Second-generation PLINK: rising to the challenge of larger and richer datasets. *Gigascience*. 2015;**4**:7.

14. Graham BL, et al. 2017 ERS/ATS standards for single-breath carbon monoxide uptake in the lung. *Eur Respir J*. 2017;**49**(1).

15. Meier MH, et al. Associations Between Cannabis Use and Physical Health Problems in Early Midlife: A Longitudinal Comparison of Persistent Cannabis vs Tobacco Users. *JAMA Psychiatry*. 2016;**73**(7):731-40.

16. Thomson WM, Shearer DM, Broadbent JM, Foster Page LA, Poulton R. The natural history of periodontal attachment loss during the third and fourth decades of life. *J Clin Periodontol*. 2013;**40**(7):672-80.

17. Reuben A, et al. Lest we forget: comparing retrospective and prospective assessments of adverse childhood experiences in the prediction of adult health. *J Child Psychol Psychiatry*. 2016;**57**(10):1103-12.

18. Beckley AL, et al. The Developmental Nature of the Victim-Offender Overlap. *J Dev Life Course Criminol*. 2018;**4**(1):24-49.

19. Carlin JB, Gurrin LC, Sterne JA, Morley R, Dwyer T. Regression models for twin studies: a critical review. *Int J Epidemiol*. 2005;**34**(5):1089-99.

**Supplementary Figures**

**Supplementary Figure S1.** Flow chart of QC procedures for DNA methylation and sample numbers in the Dunedin and E-Risk Studies.

**
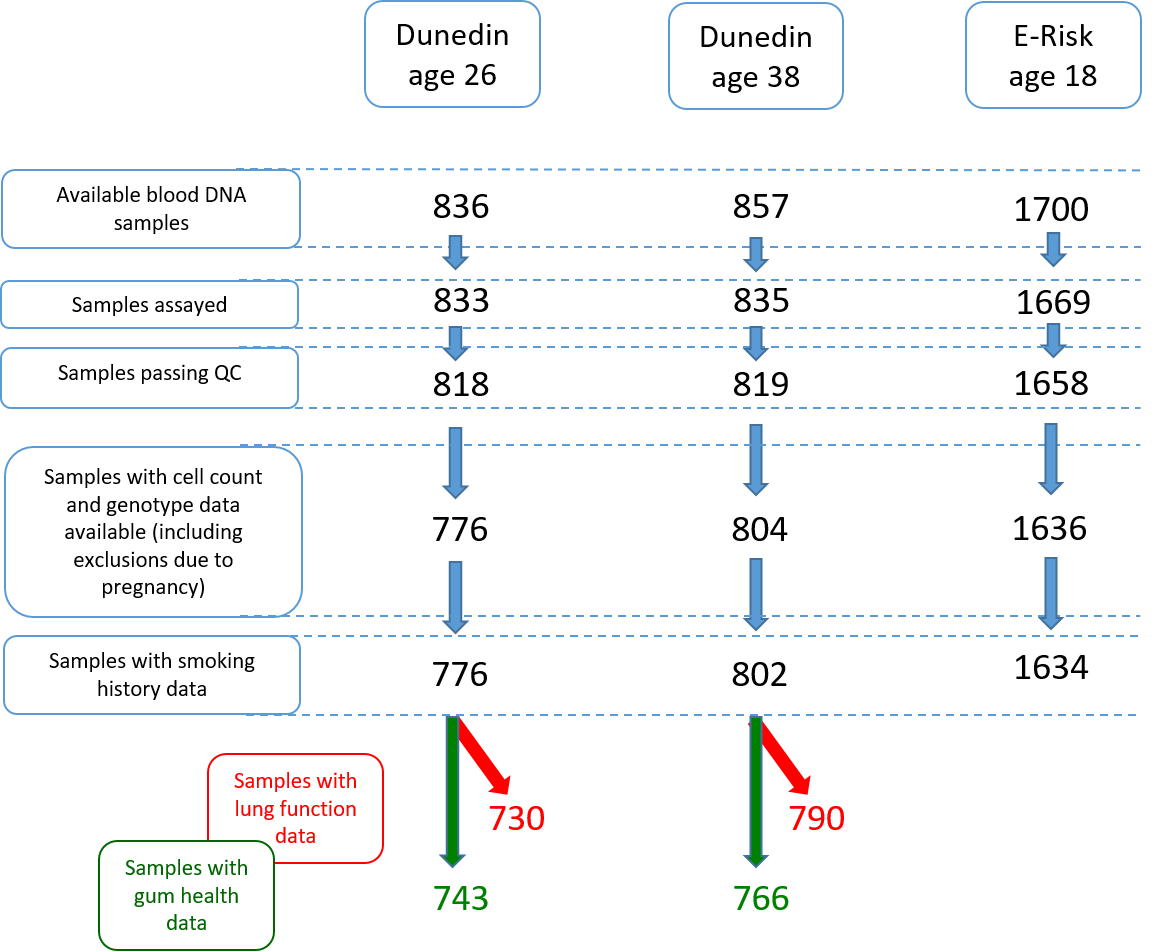
**

**Supplementary Figure S2.** ROC curves for discrimination of never- from both current- and ever-smokers by SmPEGS in Dunedin at age 38 (**Panel A**) and E-Risk at age 18 (**Panel B**). Values of AUC for each curve are shown on the chart. Improved discrimination is reflected in larger AUC for the Dunedin Study, presumably a reflection of the 20 years longer smoking history over the E-Risk participants.

**A B**


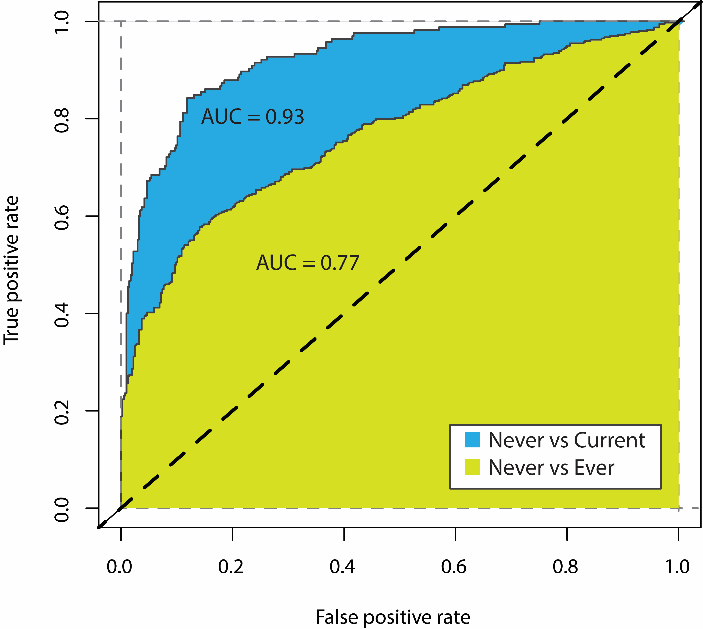

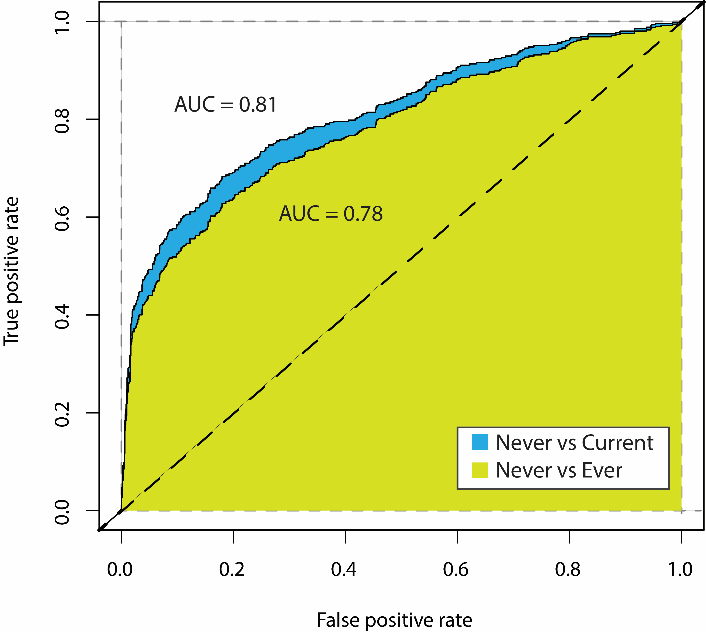


**Supplementary Figure S3.** Distribution of SmPEGS in the Dunedin Study at two time points across 12 years as a function of nicotine dependence status (assessed via FTND). The figure shows SmPEGS were reduced for those who were dependent at age 26 but not 38, whilst those who were dependent either at age 38 or at both ages saw increases in their SmPEGS. Black points represent means within each group connected by lines across the two assessment phases.


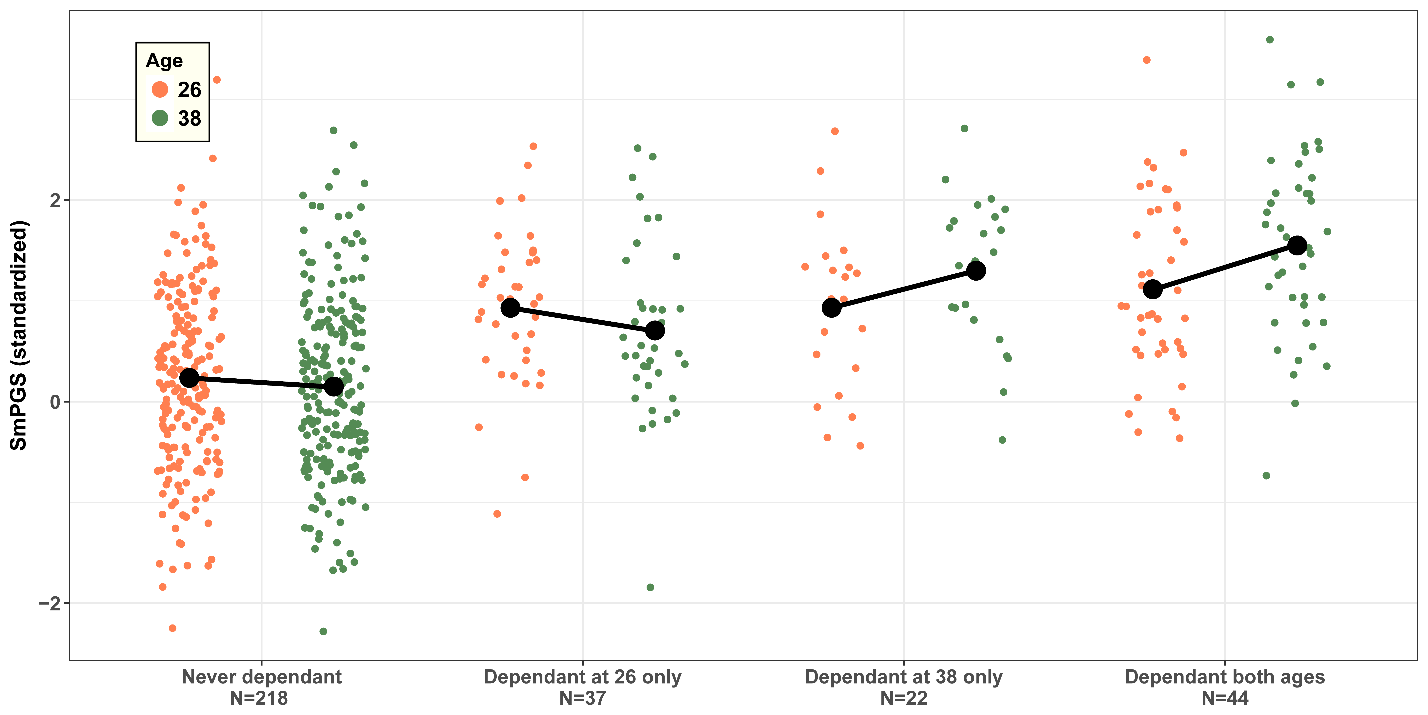

Supplement: Supplementary file 1 — Supplementary Material. [file 41398_2019_430_MOESM1_ESM.docx]
